# *IDH1*-mutated transgenic zebrafish lines: An *in-vivo* model for drug screening and functional analysis

**DOI:** 10.1371/journal.pone.0199737

**Published:** 2018-06-28

**Authors:** Ya Gao, Maurice de Wit, Eduard A. Struys, Herma C. Z. van der Linde, Gajja S. Salomons, Martine L. M. Lamfers, Rob Willemsen, Peter A. E. Sillevis Smitt, Pim J. French

**Affiliations:** 1 Department of Neurology, Erasmus Medical Center, Rotterdam, the Netherlands; 2 Department of Clinical Chemistry, VU University Medical Center, Amsterdam, the Netherlands; 3 Department of Genetics, Erasmus Medical Center, Rotterdam, the Netherlands; 4 Department of Neurosurgery, Erasmus Medical Center, Rotterdam, the Netherlands; University of Colorado Boulder, UNITED STATES

## Abstract

**Introduction:**

The gene encoding isocitrate dehydrogenase 1 (*IDH1*) is frequently mutated in several tumor types including gliomas. The most prevalent mutation in gliomas is a missense mutation leading to a substitution of arginine with histidine at the residue 132 (R132H). Wild type IDH1 catalyzes oxidative decarboxylation of isocitrate to α-ketoglutarate (α-KG) whereas mutant IDH1 converts α-KG into D2-hydroxyglutarate (D2HG). Unfortunately, there are few *in vivo* model systems for *IDH*-mutated tumors to study the effects of *IDH1* mutations in tumor development. We have therefore created transgenic zebrafish lines that express various *IDH1* mutants.

**Materials and methods:**

*IDH1* mutations (*IDH1*^*R132H*^, *IDH1*^*R132C*^ and loss-of-function mutation *IDH1*^*G70D*^), *IDH1*^*wildtype*^ or *eGFP* were cloned into constructs with several brain-specific promoters (*Nestin*, *Gfap* or *Gata2)*. These constructs were injected into fertilized zebrafish eggs at the one-cell stage.

**Results:**

In total more than ten transgenic zebrafish lines expressing various brain-specific *IDH1* mutations were created. A significant increase in the level of D2HG was observed in all transgenic lines expressing *IDH1*^*R132C*^ or *IDH1*^*R132H*^, but not in any of the lines expressing *IDH1*^*wildtype*^, *IDH1*^*G70D*^ or *eGFP*. No differences in 5-hydroxymethyl cytosine and mature collagen IV levels were observed between wildtype and mutant IDH1 transgenic fish. To our surprise, we failed to identify any strong phenotype, despite increased levels of the oncometabolite D2HG. No tumors were observed, even when backcrossing with *tp53*-mutant fish which suggests that additional transforming events are required for tumor formation. Elevated D2HG levels could be lowered by treatment of the transgenic zebrafish with an inhibitor of mutant IDH1 activity.

**Conclusions:**

We have generated a transgenic zebrafish model system for mutations in *IDH1* that can be used for functional analysis and drug screening. Our model systems help understand the biology of *IDH1* mutations and its role in tumor formation.

## Introduction

Somatic missense mutations in the gene encoding isocitrate dehydrogenase 1 (*IDH1*) or *IDH2* are frequently identified in various malignancies including gliomas, acute myeloid leukemia, cholangiocarcinoma, chondrosarcoma and sporadically in various other cancer types [1–8]. In gliomas, *IDH1* mutations are one of the earliest genetic changes identified, preceding other common genetic aberrations such as *1p19q* co-deletion, and are therefore present in virtually all tumor cells [9–11]. *IDH1* and *IDH2* mutations are almost always mutually exclusive. For glioma patients, presence of IDH mutations is of clinical relevance as patients harboring *IDH* mutated gliomas have a better survival compared to those with wildtype *IDH*. The prognostic significance of *IDH* mutations has led to its incorporation in the WHO 2016 update to classify gliomas [12]. Mutations in *IDH1* are almost always heterozygous point mutations affecting the arginine at position 132 (R132). Approximately 90% of these mutations in gliomas are *IDH1*^*R132H*^.

Wildtype IDH1 is a cytoplasmic enzyme that catalyzes the oxidative decarboxylation of isocitrate to α-ketoglutarate (αKG) and uses NADP^+^ as a co-factor [13, 14]. The mutant enzyme however, uses αKG as a substrate to produce D-2-hydroxyglutarate (D2HG) with concomitant consumption of NADPH [15]. The resulting accumulation of D2HG then competitively inhibits a spectrum of αKG-dependent enzymes including TET2, JMJD2 and various prolyl hydroxylases [16–18]. This inhibition ultimately facilitates carcinogenesis by retaining cells in an undifferentiated and stem-like state. Because of the oncogenic role of mutant IDH1, several groups have developed compounds that specifically inhibit the activity of the mutant enzyme [19, 20]. These inhibitors are currently being tested in clinical trials.

Several *IDH1*^*R132H*^ conditional knock-in (KI) mouse models were recently generated to further study the role of the mutant enzyme in an *in vivo* model system. Unfortunately most mice in which IDH1 mutations were conditionally expressed either died before birth or rapidly after induction of expression of the mutant enzyme [21]. Nevertheless, expression of mutant IDH1 results in a retention of cells in an undifferentiated state or induces pre-cancerous lesions in cartilage or the SVZ [22–24]. Despite these signs of early tumor formation, no gliomas in any of the three mouse models were thus-far identified, also not when backcrossing into a *Tp53* -mutant background [23, 25].

To further study the effects of IDH1 mutations in tumor development, we have generated transgenic zebrafish that express IDH1 mutants under the control of various CNS-specific promoters.

## Materials and methods

### Cloning

Human *pEGFP-IDH1*^*wildtype*^ and *pEGFP-IDH1*^*R132H*^ constructs were described as previous [26]. *IDH1*^*R132C*^ and *IDH1*^*G70D*^ mutations were cloned by in-fusion PCR with two sets of primers, 5’-CTATCATCATAGGTTGTCATGCTTATGGGGATCAATAC-3’ and 5’-CCATAAGCATGACAACCTATGATGATAGGTTTTAC-3’ for *IDH1*^*R132C*^; 5’-AGAAGCATAATGTTGACGTCAAATGTGCCAC-3’ and 5’-GTGGCACATTTGACGTCAACATTATGCTTCT-3’ for *IDH1*^*G70D*^. The whole construct was linearized and inserted into a miniTol2 vector (Addgene, plasmid #31829).

### Generation of transgenic zebrafish

All experiments with zebrafish (Tupfel long fin or TL) were conducted according to the protocols approved by the Animal Experimentation Committee of the Erasmus Medical center and EU guidelines. To generate transgenic zebrafish lines expressing *GFP-IDH1*^*wildtype*^ and *GFP-IDH1*^*R132H*^ driven by different promoters (*Nestin*, *Gata2* and *Gfap*), we injected various constructs into the cells of fertilized zebrafish eggs at the one-cell stage. Embryos that showed GFP expression at 1 day post fertilization (dpf) were collected and raised to adulthood (3 months, F0) and then individually crossed with non-transgenic wildtype TL (F1). GFP expression in the F1 fish indicated that the constructs were integrated into the fish genome. The GFP-positive F1s were separately raised to adults and then interbred to generate homozygous F2. Although we did not actually test for homozygosity of our transgenic lines, we inferred this by the observation that all F2 inbred offspring expressed GFP. All F2 progenies were further inbred. The experiments were performed mainly on likely homozygous F4 zebrafish. *tp53* mutant fish (*tp53*^M214K^) were described by Berghmans et al and obtained from ZFIN (ZFIN.org) [27].

### Histology and Immunohistochemistry

Zebrafish embryos were fixed overnight in 4% paraformaldehyde (PFA) at 4°C and then embedded in paraffin for further histological analysis. Paraffin sections (6 μm) were stained with hematoxylin and eosin (HE). For immunohistochemistry (IHC), paraffin sections were dewaxed and hydrated followed by boiling in 10 mM sodium citrate for eight minutes and 2 times 3 minutes of boiling in a microwave oven. Prior to immunostaining, the endogenous peroxidase activity was blocked by 30% hydrogen peroxide and 12.5% sodiumazide in PBS for 30 minutes. The slides were washed in PBS and PBS+ which contained 0.5% g/ml protifar and 0.15% g/ml glycine and then incubated with primary antibody overnight at 4°C. The primary antibodies used were anti-GFP (1:2000) monoclonal antibody (Roche, Woerden, the Netherlands), 5hmC (1: 200, Active Motif, La Hulpe, Belgium) and anti-human IDH1^R132H^ (1:200) monoclonal antibody (Dianova, clone DIA H09, Huissen, the Netherlands), diluted in PBS+. The sections were then washed in PBS+ and incubated with BrightVision Poly-HRP-Anti IgG (immunologic) for 60 minutes at room temperature (RT). The slides were washed in PBS+ and PBS and then treated with 1:50 diluted DAB-substrate (DAKO Liquid DAB substrate-chromogen system) for 4–8 minutes, followed by counterstaining with haematoxylin and mounted in entellan. Histological images were captured using an Olympus BX40 camera.

### Real-time PCR

To examine the expression of *IDH1* and *GFP* during envelopment, total RNA was extracted by dissolving embryos in 500 μl TRIzol^®^ (Life technologies, Carlsbad, USA) and 100 μl chloroform followed by centrifugation at 12.000 g for 15 min at 4°C. RNA in the aqueous phase was precipitated with 250 μl isopropanol and collected at 12 000 g for 10 minutes at 4°C. The pellet was washed twice in 250 μl 75% ethanol, centrifuged at 12 000 g for 5 minutes at 4°C, dried and dissolved in 10 μl nuclease free water (Ambion, Thermo Scientific, Rochester, USA). For cDNA synthesis, each reaction contained 1000 ng RNA, 1 μl hexamers, 1 μl 10 mM dNTP’s and milliQ water to 13 μl and was heated to 65°C for 5 minutes and left on ice for at least 1 minute. The RNA was then treated with 4 μl 5x Firststrand buffer, 1 μl 0.1M DTT, 0.5 μl RNaseOUT and 0.5 μl DNase. The samples were heated to 37˚C for 40 minutes and further heated to 65°C for 10 minutes. The RNAs were then reverse-transcribed by adding 1 μl Superscript III (Invitrogen, Breda, the Netherlands) and 0.5 μl RNase OUT followed by incubation at 25˚C for 5 minutes and 42°C for one hour. 1 μl cDNA was used in a 15 μl reaction containing 7.5 μl Syber Select Mastermix (Life technologies), 1mM primers and MilliQ. The primer sequences are described in S1 Table. The reactions were performed in triplicate using a CFX96 Real-Time PCR System (Bio-Rad). The threshold cycle (Cq) for each reaction was obtained and the values were averaged. The standard deviation (SD) had to be below 0.2. The relative expression levels, of different time points in zebrafish life, were calculated. First the ΔCq of a sample was calculated; ΔCq = *IDH1* Cq mean- *β-actin* Cq mean. Then one time point was set as a reference (= 1.00) and the ΔΔCq was calculated as ΔΔCq = ΔCq reference—ΔCq unknown sample. To calculate the relative expression levels the formula 2^ ^ΔΔCq^ was used.

### Protein extraction and immunoblotting

Zebrafish embryos were lysed in 500 μl HEPES-buffer containing 1x protease inhibitor (cOmplete, Thermo Scientific) and 3 μM DTT followed by homogenization by a PRO 200 homogenizer and incubated on ice for 30 minutes. 50 μg of protein was separated by loading onto a precast SDS gel (Thermo Scientific) and electrophoresis at 150 V till loading buffer reached the bottom of the gel. Protein was then transferred to a nitrocellulose membrane in transfer buffer at 100 V, 380 mA, for 1 hour. The membrane was blocked with blocking buffer containing 1% BSA in PBS for 1 hour at room temperature and incubated with primary antibody overnight at 4°C with agitation. Primary antibodies used were anti-Collagen Type IV (1:1000, Abcam, Hilversum, The Netherlands) and 5hmC (1: 1000).

### Microinjection of wildtype zebrafish embryos with *Gfap* constructs

20 μl of injection solution containing 350 ng of *Gfap* constructs, 30 ng/μl Tol2 transposase RNA and 0.1% pheno-red was freshly prepared before injection. 4.2 nl of injection solution was injected into the cell of 1-cell stage embryos using a Pneumatic PicoPump (PV820, WPI). For each construct, injection was performed on 100 eggs in three independent experiments. The fertilization rate was calculated based on 30 uninjected embryos collected on the same day. The number of GFP^+^, GFP^-^, healthy and abnormal embryos were counted on 1dpf.

### 5hmC assay

Total DNA was extracted using whole fish embryos. A nitrocellulose membrane was pre-soaked in 20X SSC for 1 hour. 250 ng DNA was diluted in 150μl H2O and 150μl 20X SSC. The membrane and two layers of thick filter papers were placed on a manifold (manifold II slot-blot manifold, Cole-Parmer, Wertheim, Germany) and equilibrated with 10X SSC. DNA samples were then loaded and fixed on the membrane using a vacuum pump for 5 minutes. The membrane was air-dried and processed as described above in the immunoblotting section. Blots were stained using the 5hmC antibody (1:1000) and analyzed using ECL.

### IDH1 mutant inhibitor test

Five transgenic zebrafish embryos from each line were screened for GFP expression at 1 or 2 dpf and removed from the chorion and raised in 2 ml tap water for 48 hours with either 10μM AGI-5198 (Xcess Biosciences, Inc.) in 0.1% DMSO or 0.1% DMSO. Zebrafish embryos were collected at 3 dpf in 25 μl of HBSS buffer for 2HG measurement.

### Quantification of D/L2HG in zebrafish

To quantify the level of D- and L2HG in the zebrafish, five embryos were collected at 1, 2, 3, 5 and 6 dpf in HBSS buffer (5μl per embryo). The embryos were homogenized with a PRO 200 homogenizer and lysed with sonication before LC-MS/MS. The D and L forms of 2HG were separately measured and quantified as described previously [28].

### Statistics

Differences in D- and L- 2HG levels between experimental conditions were evaluated using the students t-test. A Fisher’s exact test was used to compare differences between frequencies.

## Results

### Generation and characterization of transgenic zebrafish lines

We firstly generated transgenic lines for two constructs, *eGFP-IDH1*^*wildtype*^ and *eGFP-IDH1*^*R132H*^, in which the transgene was expressed under control of a *Nestin* promoter. These constructs are referred to as *Nes*^*IDH1wt*^ and *Nes*^*R132H*^. At least two independent lines per construct were generated to control for integration-site dependent effects.

**Fig 1:**
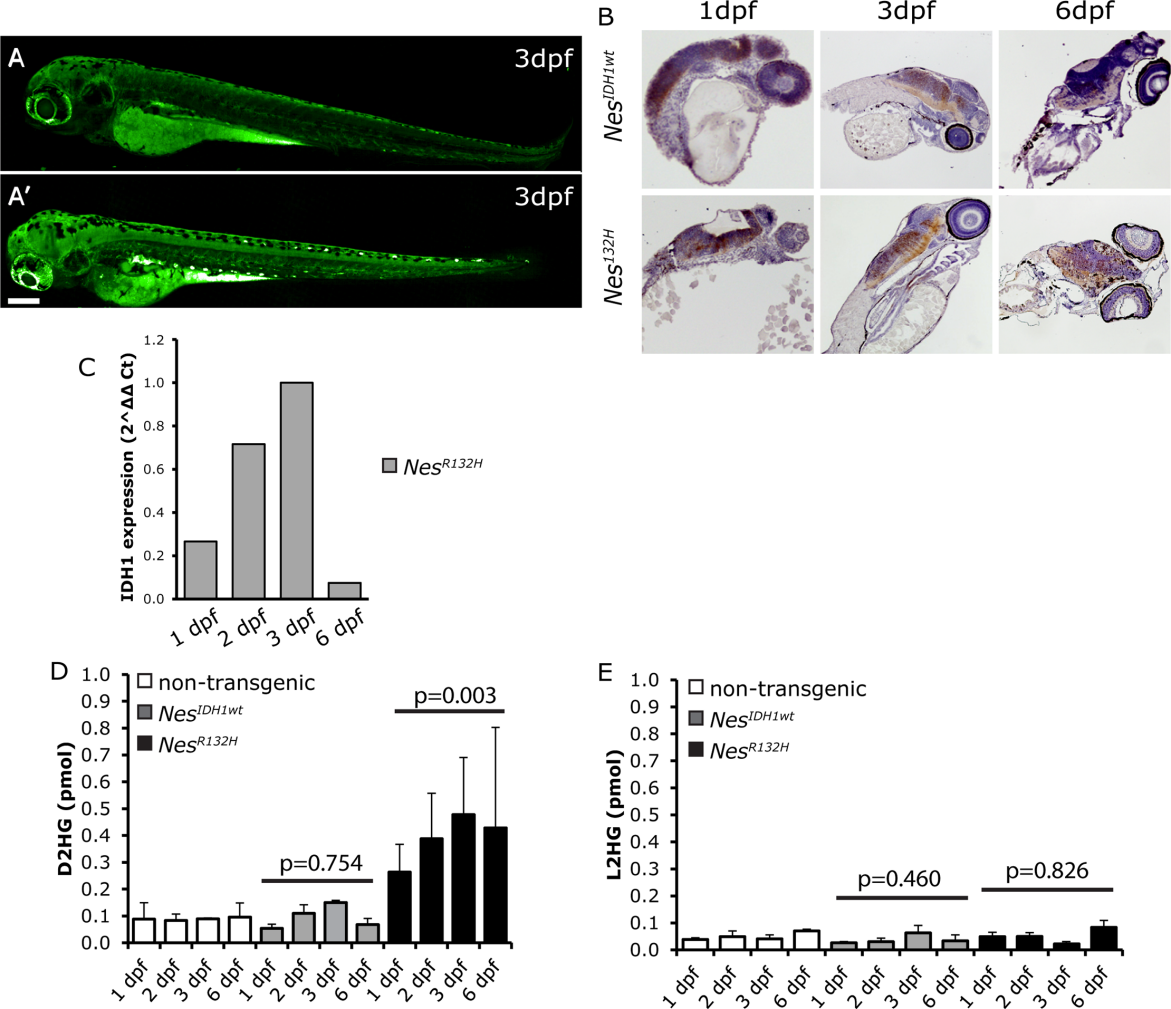
Characterization of *Nes*^*IDH1*^ zebrafish lines. Expression of the transgene was detected in the central nervous system (CNS) of zebrafish embryos using fluorescent imaging (A: non-transgenic wildtype zebrafish showing background auto-fluorescence staining, mainly in the yolk sac; A’: *Nes*^*IDH1*^ show expression of the transgene in the CNS of 3dpf embryos). Expression was confirmed by immunochemistry staining using an anti-GFP antibody (B) and Q-PCR (C). D2HG only accumulated in *Nes*^*R132H*^ zebrafish (D, non-transgenic vs *Nes*^*IDH1wt*^, p = 0.754, non-transgenic vs *Nes*^*R132H*^, p = 0.003, student’s t-test). L2HG levels in the transgenic lines showed no such increase (E). For Q-PCR experiments, we used a pool of five fish per time-point; D2HG and L2HG measurements were averages of two replicates using 5 fish per replicate. Scale bar: 200 μm.

Transgene expression was detected in the brain and spinal cord on 1, 3 and 6 days post fertilization (dpf) by fluorescent imaging (Fig 1A and S1A Fig) and by immunohistochemistry (Fig 1B) using anti-GFP antibodies. Expression of *Nes*^*R132H*^ was confirmed using an IDH1^R132H^-mutant specific antibody (S2 Fig). As expected, this antibody did not show staining in the *Nes*^*IDH1wt*^*-*fish. Expression of transgenes on 1, 2, 3 and 6 dpf was also detected on the RNA level by RT-QPCR (Fig 1C). We then measured D2HG levels to monitor the activity of the neomorphic enzyme. Consistent with RNA and protein expression, the D2HG level in *Nes*^*R132H*^ mutant transgenic fish was elevated compared to controls (non-transgenic and *Nes*^*IDH1wt*^) on 1–5 dpf (Fig 1D). The increase in D2HG was virtually identical when using macro-dissected embryos (head region) compared to whole fish (S3 Fig). L2HG levels in all the transgenic lines were similar to the non-transgenic controls (Fig 1E); indicating expression of *IDH1*^*R132H*^ only affects D2HG levels. D2HG levels returned to normal by 21 dpf. These experiments demonstrate CNS-specific expression of *IDH1*^*wt*^ or *IDH1*^*R132H*^ in our transgenic zebrafish lines during development. This temporal expression pattern in the CNS is consistent with the *Nestin* promoter activity [29–31].

It has been reported that accumulation of D2HG results in DNA hypermethylation by inhibition of TET enzymes [32]. In our transgenic fish, DNA methylation as determined by 5hmC antibody staining was however not affected (S4 Fig). We next screened for collagen maturation defects, as these were observed in an IDH1^R132H^-KI mouse model [21]. However, western blot analysis failed to detect the presence of immature isoforms of collagen in our transgenic fish lines (S5 Fig). In summary, despite expression of the transgene (and the elevated levels of D2HG in lines expressing *Nes*^*R132H*^), all of the zebrafish lines remained healthy without presenting any identifiable developmental abnormalities (S6 Fig).

Given the short temporal expression of *IDH1* constructs driven by the *Nestin* promoter, we cloned constructs under the control of a brain specific *Gata2* promoter. This promoter was previously used for constructing a transgenic zebrafish model for neurodegeneration [33]. Three transgenic lines were generated, *pGata2-GFP-IDH1*^*wt*^, *pGata2-GFP-IDH1*^*R132H*^ and *pGata2-GFP*. These constructs are referred to as *Gata*^*IDH1wt*^, *Gata*^*R132H*^ and *Gata*^*GFP*^. Unfortunately, we failed to observe any transgene expression in the developing (or adult) CNS in any of the lines generated. We did however observe expression in the notochord from 1 for up to 5 dpf (S7 Fig), but, despite expression of a D2HG-producing IDH1 mutant, all fish were viable, developed normally and produced offspring. Similar to the *Nes*^*IDH1wt*^ and *Nes*^*R132H*^ fish, no gross abnormalities or (pre-) neoplastic lesions were observed. As we failed to observe expression in the CNS we did not further investigate these lines.

**Fig 2:**
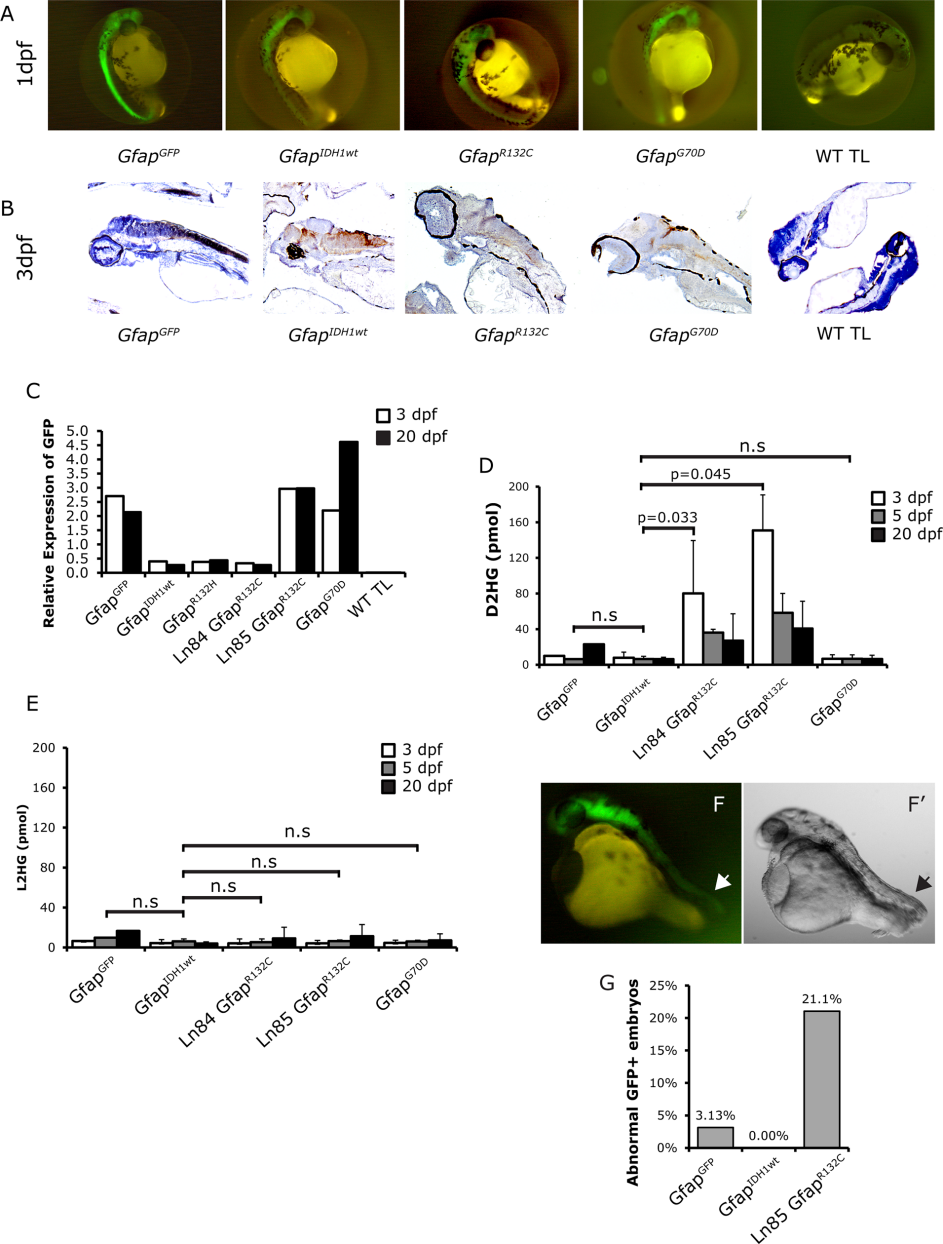
Characterization of *Gfap* zebrafish lines. Expression of transgene was detected using fluorescent imaging (A), immunohistochemistry with an anti-GFP antibody (B) and QPCR (C). Elevated levels of D2HG were only detected in *Gfap*^*R132C*^ lines (D). L2HG levels in the transgenic fish embryos were not affected (E). About 21% *Gfap*^*R132C*^ embryos showed defects in tail development on 1dpf (F and G). Arrow heads: the site of abnormal tail development in the *Gfap*^*R132C*^ embryos. For Q-PCR experiments, we used a pool of five (3dpf) or three (20 dpf) fish per time-point; D2HG and L2HG measurements were averages of two replicates using five (3 and 5 dpf) or three (20 dpf) fish per replicate. Scale bar: 500 μm.

Because of the temporal limitations of the *Nestin* promoter and the lack of expression in the CNS using the *Gata2* promoter, we generated six additional lines, one for *eGFP-IDH1*^*wildtype*^, *eGFP-IDH1*^*R132H*^, *eGFP-IDH1*^*G70D*^ and e*GFP*, and two for *eGFP-IDH1*^*R132C*^, in which the transgene was expressed under control of a *Gfap* promoter. Constructs used for these lines are referred to as *Gfap*^*IDH1wt*^, *Gfap*^*R132H*^, *Gfap*^*R132C*^, *Gfap*^*G70D*^ and *Gfap*^*GFP*^. The expression vector (*gfap*:*GFP*) has been demonstrated to have glial-specific expression in the zebrafish CNS, detectable during the embryonic stage [34]. The *IDH1*^*G70D*^ mutation is an enzymatic null mutation that was included to serve as a non-D2HG-producing control [15, 35]. The *IDH1*^*R132C*^ mutation was generated to study potential differences between *IDH1*^*R132H*^ and *IDH1*^*R132C*^, and is a mutant with reportedly higher neomorphic enzymatic activity [36]. Expression of the transgenes was confirmed using fluorescent imaging on 1, 3 and 5 dpf (Fig 2A and S1B Fig). GFP was observed in the brain and spinal cord in all the *Gfap* transgenic zebrafish lines. CNS-specific expression of the transgene was further confirmed by immunohistochemistry using anti-GFP antibodies (Fig 2B). Expression of the transgene on the RNA level was confirmed by RT-QPCR till at least 20 dpf (Fig 2C). There were no obvious differences in results from these assays between *IDH1*^*R132H*^ and *IDH1*^*R132C*^ transgenic zebrafish. The D2HG levels were markedly elevated in both *Gfap*^*R132C*^ zebrafish lines (line 84 and 85) on 3 dpf, which are about 8 and 18 times higher compared to the *Gfap*^*GFP*^ and *Gfap*^*IDH1wt*^ lines (line 73 and 92, Fig 2D). D2HG levels in *Gfap*^*G70D*^ zebrafish remained similar as in *Gfap*^*GFP*^ and *Gfap*^*IDH1wt*^ lines. L2HG levels of all the IDH1 transgenic lines were similar to the *Gfap*^*GFP*^ control, confirming that expression of transgene only affects the D2HG level (Fig 2E).When crossing *Gfap*^*R132C*^ line 85, an average of 21.1% of generated embryos showed abnormal tail development on 1dpf, which was higher than in the *Gfap*^*GFP*^ (3%) and *Gfap*^*IDH1wt*^ (0%) control lines (Fig 2F and 2G). The tail defects may be explained by the fact that the first detectable expression of *Gfap* is at 10 h post fertilization in the developing tail bud [34]. However, no clear (pre-) cancerous lesion was observed in any of the zebrafish lines studies.

It is possible that *IDH1*^*R132H/R132C*^ induced a pathologic phenotype due to the site of integration of our transgene. To correct for potential integration site artifacts, we directly injected fertilized zebrafish eggs at the one-cell stage with various constructs and monitored zebrafish development. We specifically monitored tail development in our transgenic fish. In three independent experiments, we injected fertilized eggs with *Gfap*^*GFP*^, *Gfap*^*IDH1wt*^ or *Gfap*^*R132C*^ constructs. While most embryos injected with *Gfap*^*GFP*^ remained healthy (n = 105/129, S8A and S8D Fig), most embryos injected with the *Gfap*^*R132C*^ construct were not (n = 75/85, P<0.001, Fisher’s exact test). In line with our transgenic lines, many showed an abnormal development of the tail on 1dpf. However, zebrafish embryos injected with *Gfap*^*IDH1wt*^ constructs also sometimes had abnormal tail development, though the frequency was significantly lower than that of *Gfap*^*R132C*^ (n = 13/24, P<0.001).

### tp53 deficient Transgenic zebrafish crossed with IDH1 mutant fish

*TP53* mutations often co-occur in IDH1-mutated astrocytomas. To determine whether *Gfap*^*R132C*^ affects tumor formation, we crossed the homozygous *tp53*^*M214K*^ mutant transgenic zebrafish with our transgenic zebrafish lines. It was previously reported that homozygous *tp53* mutant zebrafish developed tumors (Schwannomas) at ~8 months post fertilization with an incidence of 28% [27]. Although we find that heterozygous *tp53* mutant fish developed tumors (Table 1 and S9 Fig, incidence = 15%, n = 2/13) with an average age of onset ~1 year post fertilization, this incidence was not increased when the fish were crossed into a *pGfap*:*GFP-IDH1*^*R132H*^ (or ^wt^) background, with incidence between 6 to 14.3% regardless of the *IDH1* variants or *GFP* controls (P>0.3 for all comparisons, Fisher’s exact test). The non-CNS tumors we observe in our transgenic lines are most likely Schwannomas, as previously described [27]. They are mainly in the abdominal cavity, an area where we do not see expression of our transgene. Our results therefore demonstrate that expression of mutant IDH1 does not promote tumor formation in *tp53* mutant zebrafish.

**Table 1:** Tumorigenesis incidence of *Gfap* fish after crossing with *Tp53* mutant.

	# of generated fish	# of fish with tumor (over 1 year post fertilization)	Incidence of non-CNS-tumors (%)	Incidence of CNS-tumors (%)
*Gfap*^*GFP*^	35	3	8.6	0
*Gfap*^*R132C*^	30	3	10	0
*Gfap*^*G70D*^	28	4	14.3	0
*Gfap*^*wt*^	15	1	6	0
Heterozygous *Tp53* mutant	13	2	15	0

### IDH1 mutated transgenic zebrafish as an in vivo model for drug screening

**Fig 3:**
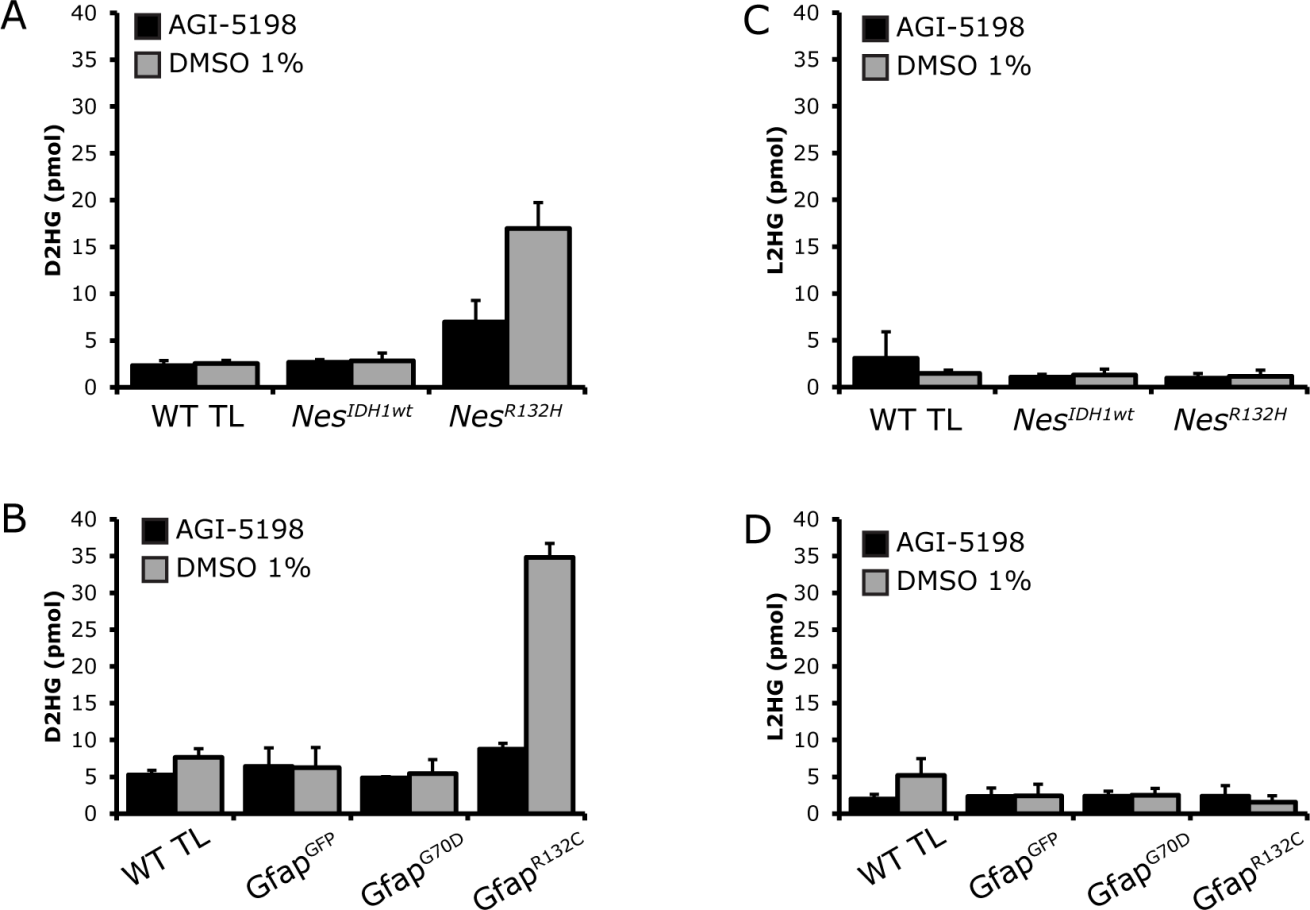
Treatment of transgenic zebrafish with 10μM IDH1 mutant inhibitor, AGI-5198, resulted in a reduction in the D2HG level in the IDH1 mutant zebrafish. D2HG level in *Nes*^*R132H*^ transgenic zebrafish was reduced to 41% of untreated (A). D2HG level in *Gfap*^*R132H*^ transgenic zebrafish was reduced to 25% of untreated (B) The L2HG level was not affected by AGI-5198 (C and D). D2HG and L2HG measurements were averages of two replicates using five fish per replicate.

AGI-5198 is a specific inhibitor for the IDH1 mutant enzymatic activity [19]. To determine whether this inhibitor also affects D2HG production *in vivo*, we applied it to our transgenic zebrafish lines harboring different IDH1 variants under control of the *Nestin* and *Gfap* promoters at 1dpf for 48 hours. Dose-response analysis indicates maximal inhibition at 10μM AGI-5198 on the *Nes*^*IDH1*^ transgenic zebrafish (S10 Fig). The inhibitor did not show any overt toxicity, even after prolonged (2 days) treatment. The accumulated D2HG in our *Nes*^*R132H*^ transgenic zebrafish was decreased by 10 μM AGI-5198 to 41% of the levels prior to treatment whereas D2HG levels in the non-transgenic and *Nes*^*IDH1wt*^ transgenic zebrafish were not affected (Fig 3A). The D2HG levels were also markedly reduced in the *Gfap*^*R132C*^ zebrafish line from 34.86 reduced to 8.77 pmol (25% of the D2HG level in the untreated fish (Fig 3B)). Levels of D2HG in control transgenic lines remained low and were not affected by the inhibitor. The L2HG level in all of the treated transgenic lines was not altered (Fig 3C and 3D). These data demonstrate that our transgenic zebrafish lines can be used to screen the efficacy and toxicity of drugs that inhibit IDH1 mutant enzyme activity.

## Discussion

Studying IDH1 mutations in gliomas has been hampered by the difficulty in generating appropriate model systems. For example, IDH-mutated gliomas are notoriously difficult to propagate *in vitro* [37] and mouse models for IDH1 mutations often have a lethal phenotype when *IDH1*^*R132H*^ is expressed at early stages of development [21, 23, 25]. Mice can survive when the mutant protein is expressed at later stages, but often with severe phenotypes (e.g. hydrocephalus). A *Drosophila* model with UAS-Idh-R195H resulted in defects in wing expansion [38]. Here we report on transgenic zebrafish model systems for *IDH1* mutations, and show that expression of *IDH1* mutations and subsequent accumulation of D2HG does not overtly affect zebrafish development in the majority of offspring. However, tail development defects were observed in a subset of offspring in one line and also following direct injection of mutant constructs in wildtype zebrafish embryos (S8 Fig). Expression of mutant, but not wildtype *IDH1* may therefore affect the cells required for normal tail development.

In contrast to the *Nestin* or GFAP-R132H KI mouse models, we did not observe any overt phenotype in any of the *Nes*^*R132H*^ transgenic zebrafish and the majority of *Gfap* transgenic fish. In mice, the brain hemorrhage phenotype is caused by collagen maturation defects (caused by inhibition of prolyl hydroxylases by D2HG) [21]. Alternatively, brain hemorrhages may be secondary to D2HG mediated coagulation defects [39]. In our transgenic lines, we failed to detect signs of collagen maturation defects or brain hemorrhage, which may provide an explanation why our fish are able to survive into adulthood. The absence of collagen maturation defects in our fish may be related to the level of D2HG accumulation in our model system, the expression level of IDH1^R132H^ in our transgenic fish, and/or to the more limited temporal expression of our constructs. D2HG accumulation may also be limited as it is likely able to diffuse out of the fish into the water of the tank. This may explain that only a modest increase in the D2HG level was detected in the IDH1-mutated fish. We failed to detect changes in 5hmc levels in the IDH1-mutated embryos which may also be caused by insufficient accumulation of local D2HG within the fish. In addition, any potential effects on fish ‘fitness’ is selected against in the process of generating transgenic lines: only healthy fish (despite elevated D2HG levels) are used to generate stable lines.

We were unable to detect CNS-specific tumors in our transgenic zebrafish lines. This is in line with data from mouse models in which brain tumors were thus far not detected. This supports the notion that *IDH1* mutation alone may be insufficient to promote tumor formation and other genetic alterations are required [40]. In most astrocytomas, *TP53* and *IDH* mutations often co-occur [41]. However, the combination of *IDH1* and *tp53* mutations did not induce gliomas in our zebrafish model. Similarly, *IDH1* mutations also did not increase tumor incidence in *Tp53* mutant mouse model, despite the observation that *IDH1* mutations induce pre-cancerous lesions such as proliferative subventricular nodules in one mouse model, but interestingly not in an almost identical other model [23, 25]. Similarly, mutations in IDH1/2 in combination with *Tp53* deficiency were insufficient to induce gliomagenesis in RCAS/tva mouse models [40]. Since mutations in *IDH* and *TP53* are the most common genetic alterations in astrocytomas, it remains unclear which additional mutations are required to induce glioma formation in our zebrafish model. Candidate genes should include oncogenic drivers ATRX and/or PDGFRA as they are present at significant frequency in lower grade gliomas. Of note, zebrafish has been appreciated as a valid model to study tumorigenesis, for example, *tp53*-mutant fish develop schwannomas and gliomas can also be generated in zebrafish by activating akt1 alone [42]. Our data also show that D2HG can be present at high levels throughout the development of zebrafish without any overt signs of pathology (although a minority of our transgenic fish did show defects in tail development). These data are in line with the observation that some D2HG aciduria patients, which have high levels of D2HG due to mutations in *IDH2* or *D2HGDH*, do not have any overt phenotype. Moreover, D2HG aciduria patients do not have an increased incidence of brain tumors [43].

In conclusion, we developed various transgenic zebrafish models with CNS-specific expression of *IDH1* mutation. We identified tail defects in a subset of IDH1-mutant fish, but we thus-far failed to identify tumors. Nevertheless, our transgenic zebrafish are a suitable model to functionally study the *IDH1* mutation *in vivo* or to use as a drug screening model.

## Supporting information

S1 Fig **Fluorescent imaging showed expression of transgene in the central nervous system of *Nestin* (A) and *Gfap* (B) transgenic zebrafish lines on 1, 3 and 5 dpf.** White arrow head: CNS-specific GFP. Yellow arrow head: auto fluorescence in the yolk sac.
(TIF)

S2 Fig Immunohistochemistry using anti-IDH1^R132H^ antibody demonstrated expression of *IDH1*^*R132H*^ specific expression in Nestin zebrafish but not in *IDH1wt* transgenic fish. (TIF)

S3 Fig D2HG measurement in *Nes*^*IDH1wt*^ and *Nes*^*R132H*^ transgenic fish. No differences in D2HG levels between macro-dissected and whole embryos were observed.
(TIF)

S4 Fig 5hmC levels was not affected by high levels of D2HG in transgenic *Nes* ^*R132H*^ mutant zebrafish. A. 5hmC levels in *Nes*^*IDH1wt*^, *Nes* ^*R132H*^ and non-transgenic zebrafish embryos were measured using slotblot stained with an 5hmC antibody (quantification of bands on the right panel). Similar results were obtained in three independent experiments one of which is shown below. B. Representative images showing 5-hmC immunostaining in *Nes*^*IDH1wt*^, *Nes* ^*R132H*^ transgenic and non-transgenic zebrafish embryo slices at 3dpf. NT: non-transgenic zebrafish.
(TIF)

S5 Fig Collagen maturation was not affected in *Nes*^*R132H*^ mutant zebrafish. Top half of the blot was stained for type IV Collagen, bottom half was stained for Tubulin (as loading control). Similar data were obtained in three independent experiments. NT: non-transgenic zebrafish. H: head of zebrafish embryos. W: whole embryo.
(TIF)

S6 Fig *Nes*^*R132H*^ transgenic zebrafish with CNS-accumulation of D2HG showed no gross histological abnormalities on 3dpf on H&E staining. (TIF)

S7 Fig Gata2GFP transgenic zebrafish shows expression in the notochord of zebrafish. Yellow arrow: In Gata2IDHwt transgenic fish the transgene is expressed in non-CNS regions (yellow arrow) whereas GfapIDH1wt transgenic fish show CNS-specific expression of transgene (white arrow). The blue arrow shows an absence of GFP signal in non-transgenic fish.
(TIF)

S8 Fig Direct injection of fertilized zebrafish embryos with *Gfap* constructs showed mutant-specific tail defects. Fluorescent imaging showed CNS-specific expression of injected construct *Gfap*^*GFP*^ (A), *Gfap*^*IDH1wt*^ (B) and *Gfap*^*R132C*^(C) and the corresponding bright-field images (A’-C’). D: the percentage of GFP-positive embryos with tail defects per construct. The ratio of injected embryos with tail defect were calculated based on results of three independent experiments (~100 eggs/construct/experiment). n.s: non-significant. Scale bar: 500μm.
(TIF)

S9 Fig An example of a schwannoma in *tp53* deficient transgenic zebrafish crossed with IDH1 transgenic fish. Euthanized 1-year old fish with a distended abdominal cavity (A). Fish were fixed in paraffin blocks (B) and sectioned slides were stained with hematoxylin/eosin for histological examination (C). D and E: enlarged images of sections in C, histological feature of tumors were consistent with the schwannomas as previously demonstrated (36).
(TIF)

S10 Fig Dose-response analysis of AGI-5198 on *Nestin* transgenic zebrafish. Maximal inhibition is reached at 10μM.
(TIF)

S1 Table Primers used for the examination of IDH1 expression levels in zebrafish by QPCR. (PDF)
