# Supplementary figures and images for: IDH1-mutated transgenic zebrafish lines: An in-vivo model for drug screening and functional analysis
Source: PLoS One. 2018 Jun 28;13(6):e0199737. doi: 10.1371/journal.pone.0199737 (PMC6023169; doi:10.1371/journal.pone.0199737)

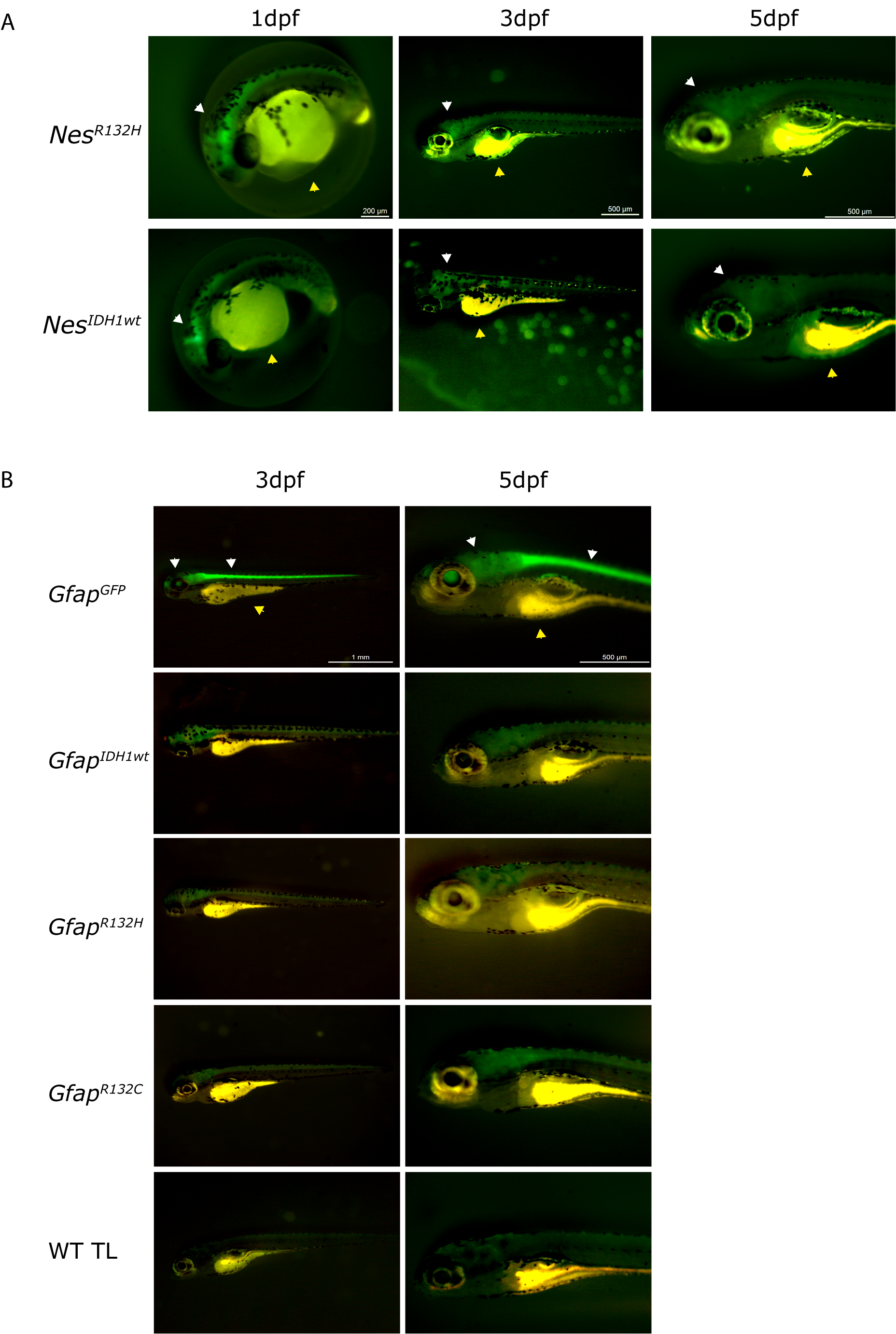

Supplement: S1 Fig — Fluorescent imaging showed expression of transgene in the central nervous system of Nestin (A) and Gfap (B) transgenic zebrafish lines on 1, 3 and 5 dpf. White arrow head: CNS-specific GFP. Yellow arrow head: auto fluorescence in the yolk sac. (TIF) [file pone.0199737.s001.tif]

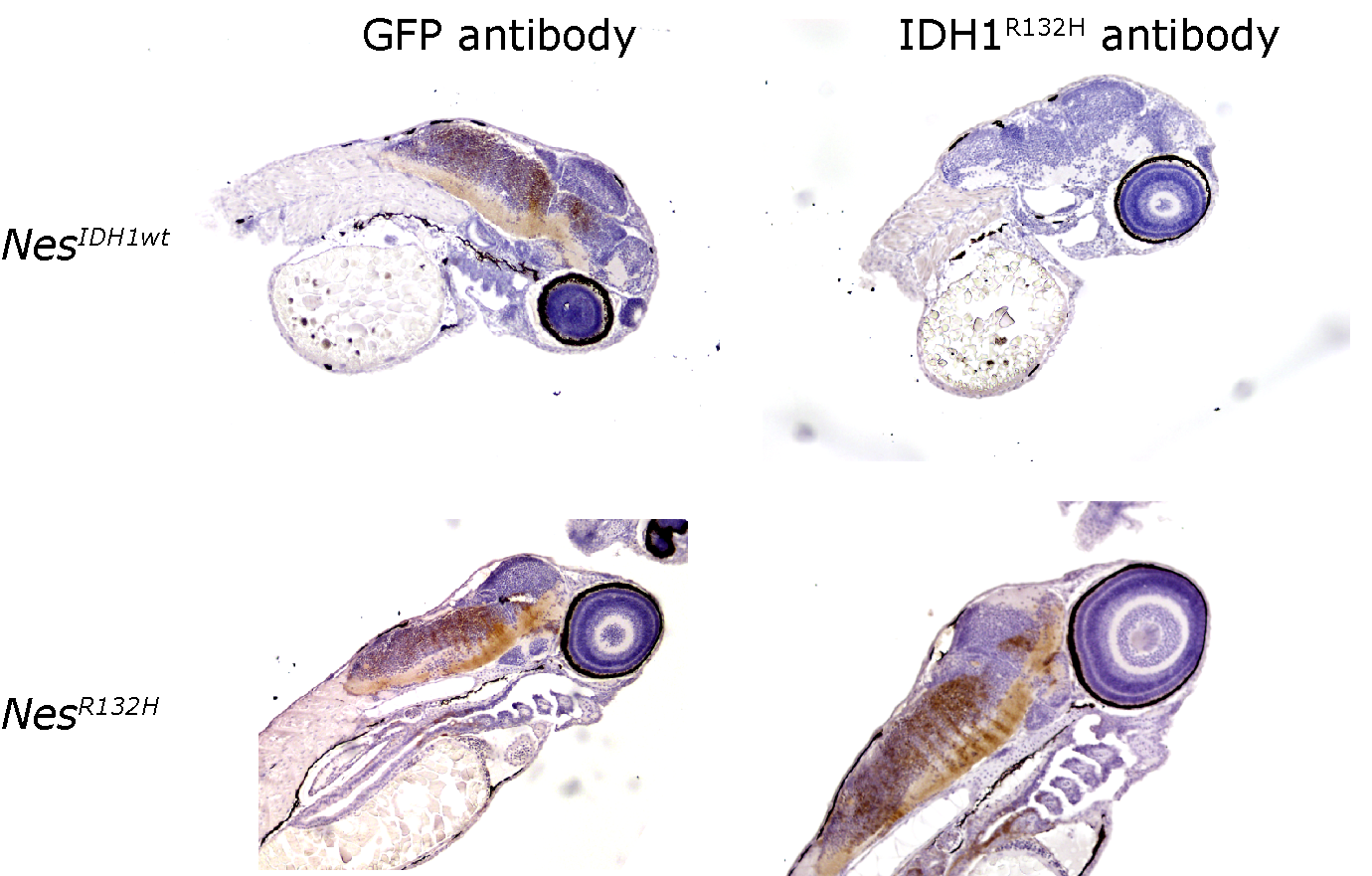

Supplement: S2 Fig — (TIF) [file pone.0199737.s002.tif]

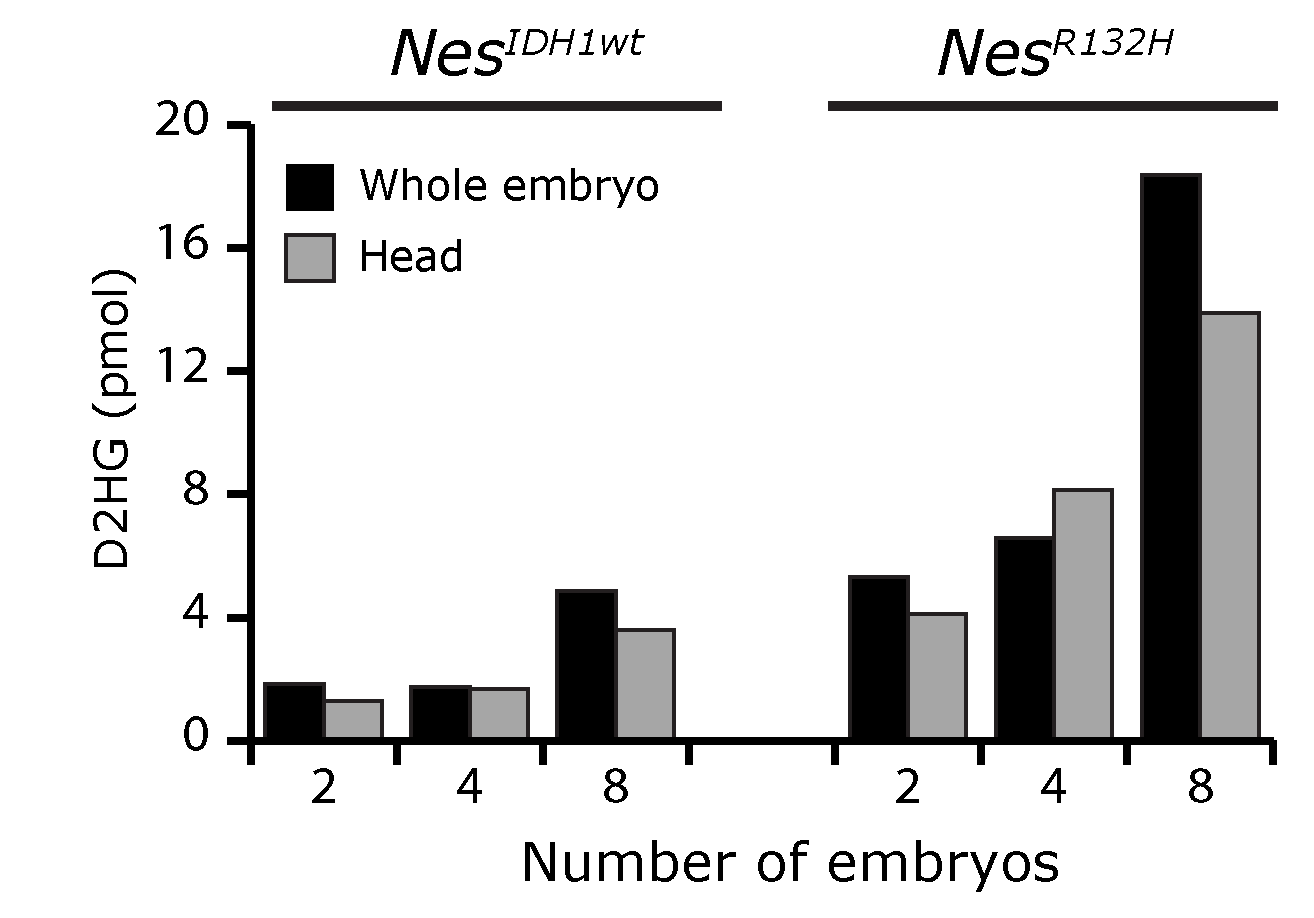

Supplement: S3 Fig — No differences in D2HG levels between macro-dissected and whole embryos were observed. (TIF) [file pone.0199737.s003.tif]

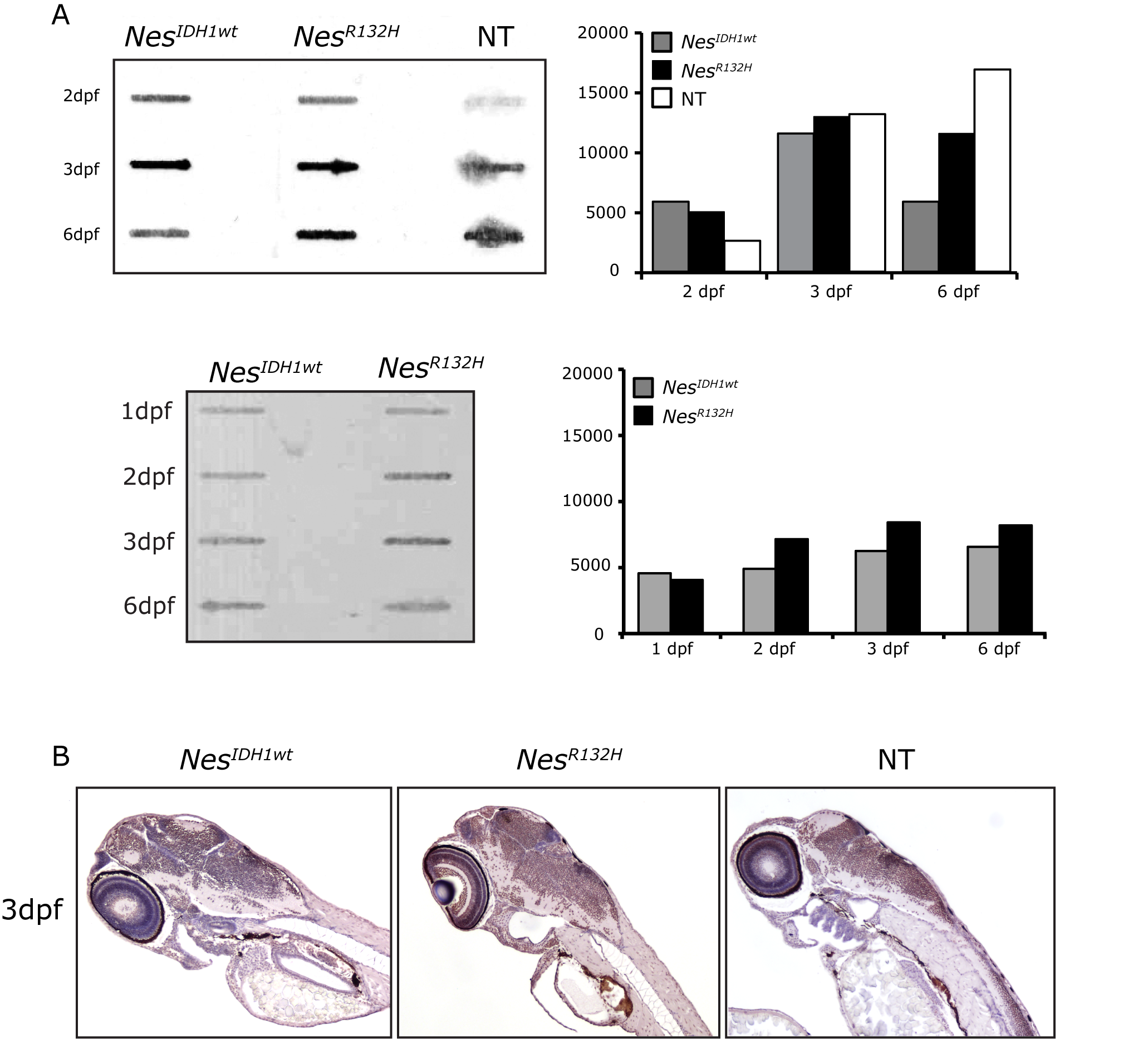

Supplement: S4 Fig — A. 5hmC levels in NesIDH1wt, Nes R132H and non-transgenic zebrafish embryos were measured using slotblot stained with an 5hmC antibody (quantification of bands on the right panel). Similar results were obtained in three independent experiments one of which is shown below. B. Representative images showing 5-hmC immunostaining in NesIDH1wt, Nes R132H transgenic and non-transgenic zebrafish embryo slices at 3dpf. NT: non-transgenic zebrafish. (TIF) [file pone.0199737.s004.tif]

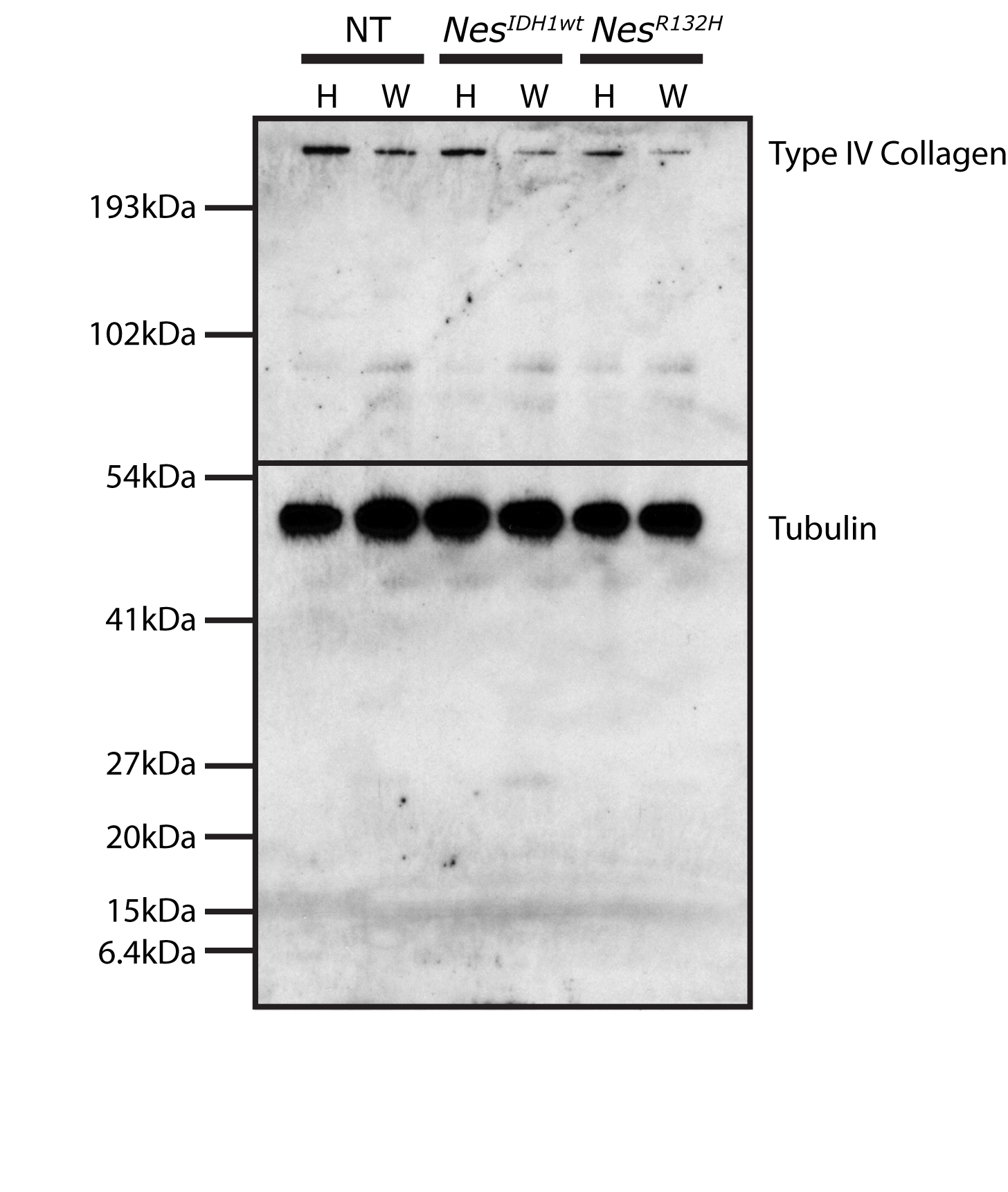

Supplement: S5 Fig — Top half of the blot was stained for type IV Collagen, bottom half was stained for Tubulin (as loading control). Similar data were obtained in three independent experiments. NT: non-transgenic zebrafish. H: head of zebrafish embryos. W: whole embryo. (TIF) [file pone.0199737.s005.tif]

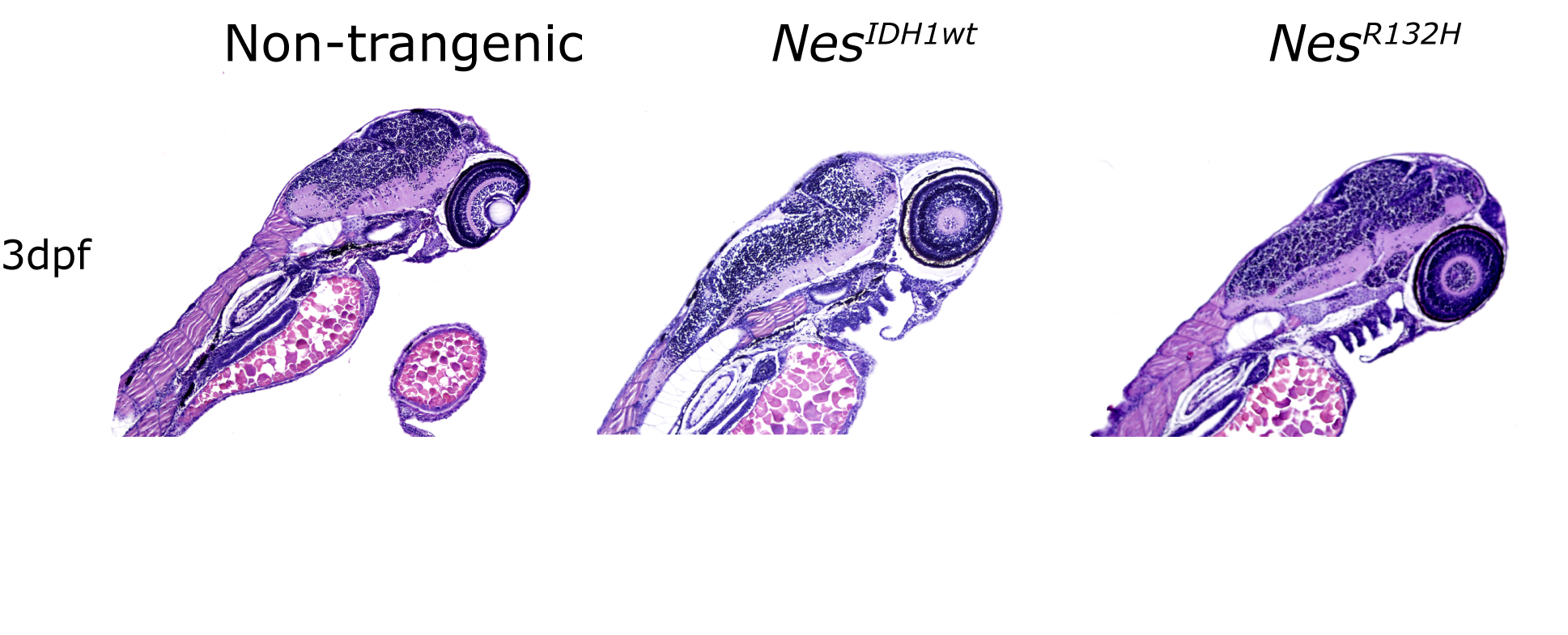

Supplement: S6 Fig — (TIF) [file pone.0199737.s006.tif]

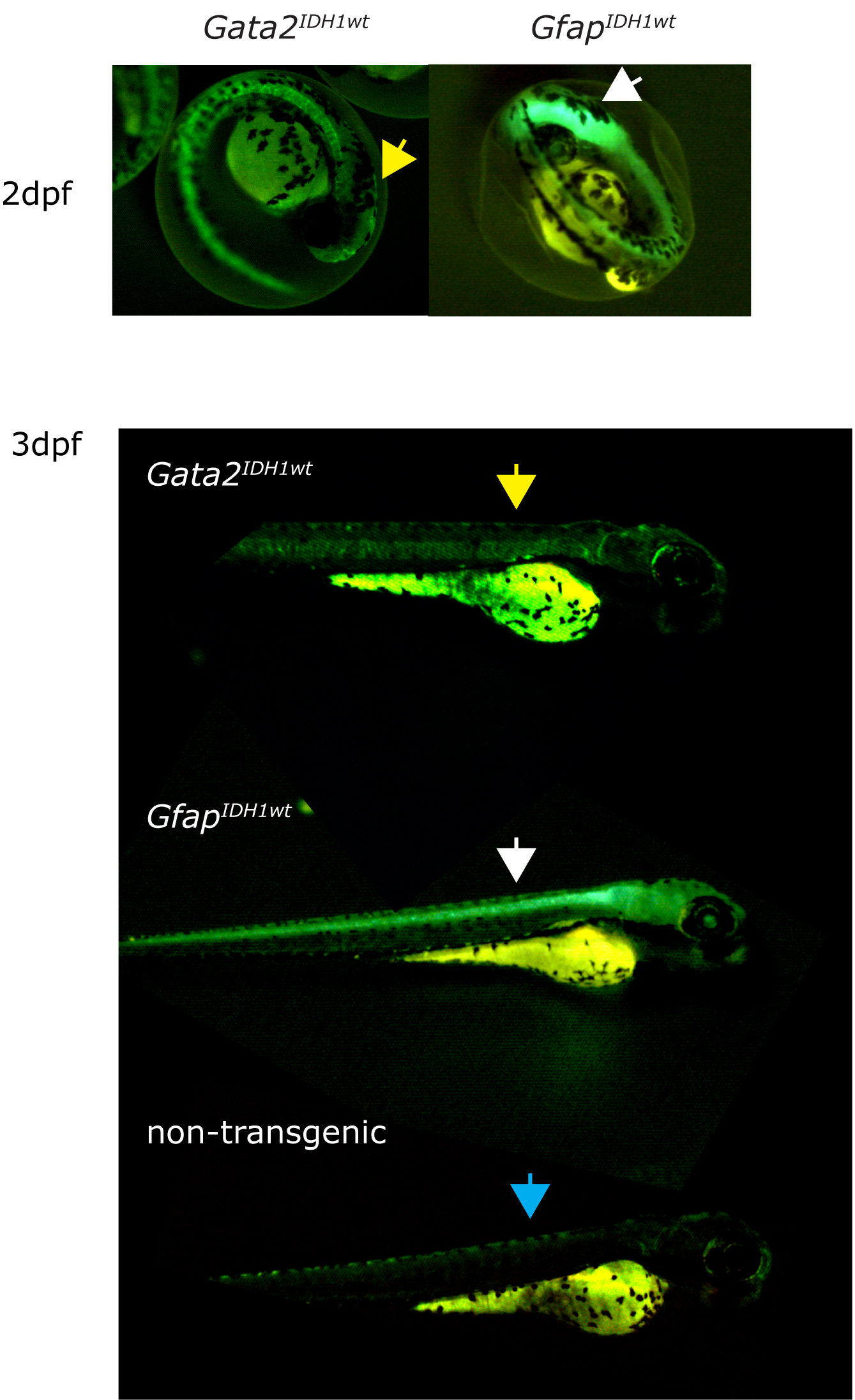

Supplement: S7 Fig — Yellow arrow: In Gata2IDHwt transgenic fish the transgene is expressed in non-CNS regions (yellow arrow) whereas GfapIDH1wt transgenic fish show CNS-specific expression of transgene (white arrow). The blue arrow shows an absence of GFP signal in non-transgenic fish. (TIF) [file pone.0199737.s007.tif]

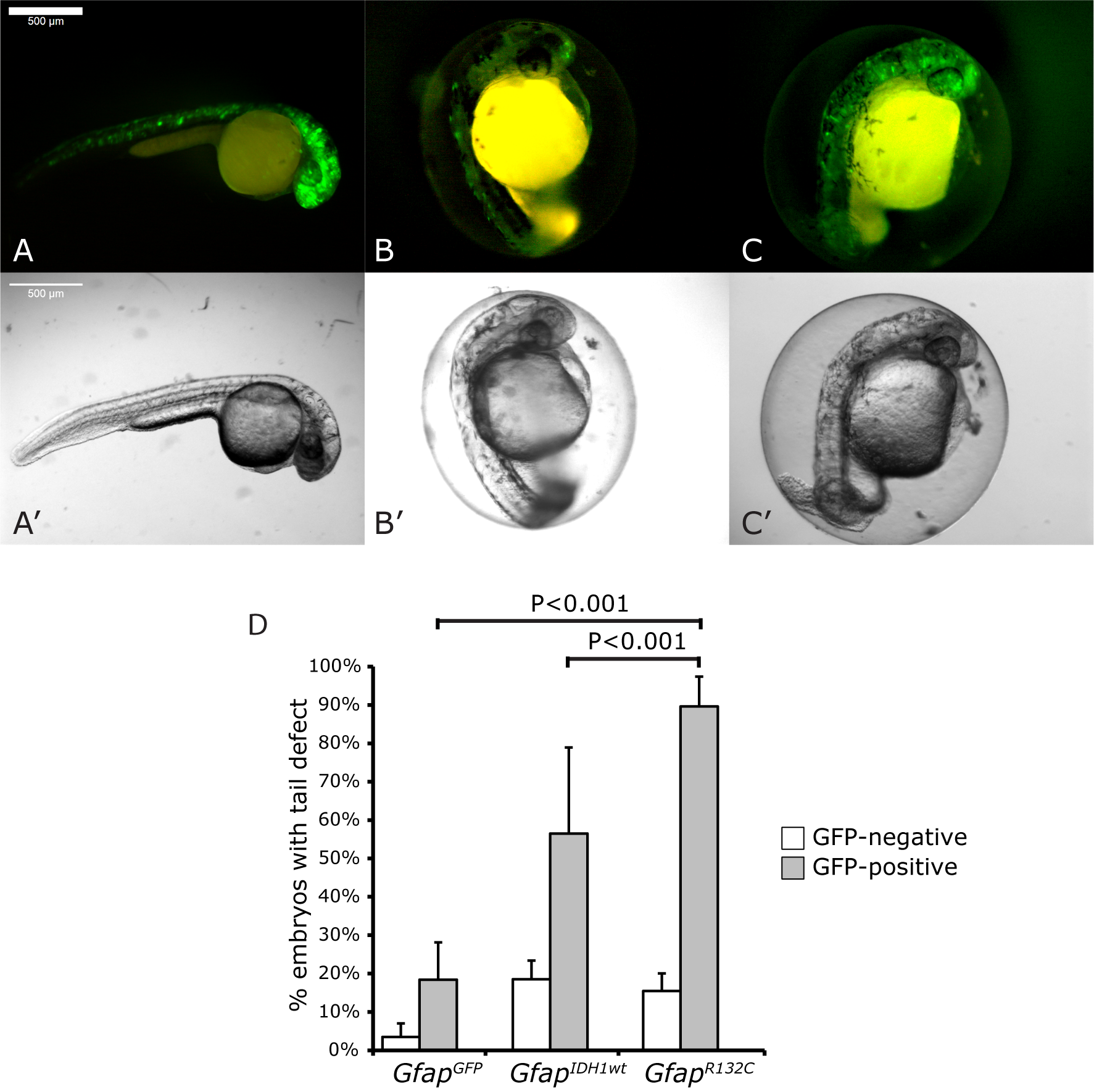

Supplement: S8 Fig — Fluorescent imaging showed CNS-specific expression of injected construct GfapGFP (A), GfapIDH1wt (B) and GfapR132C(C) and the corresponding bright-field images (A’-C’). D: the percentage of GFP-positive embryos with tail defects per construct. The ratio of injected embryos with tail defect were calculated based on results of three independent experiments (~100 eggs/construct/experiment). n.s: non-significant. Scale bar: 500μm. (TIF) [file pone.0199737.s008.tif]

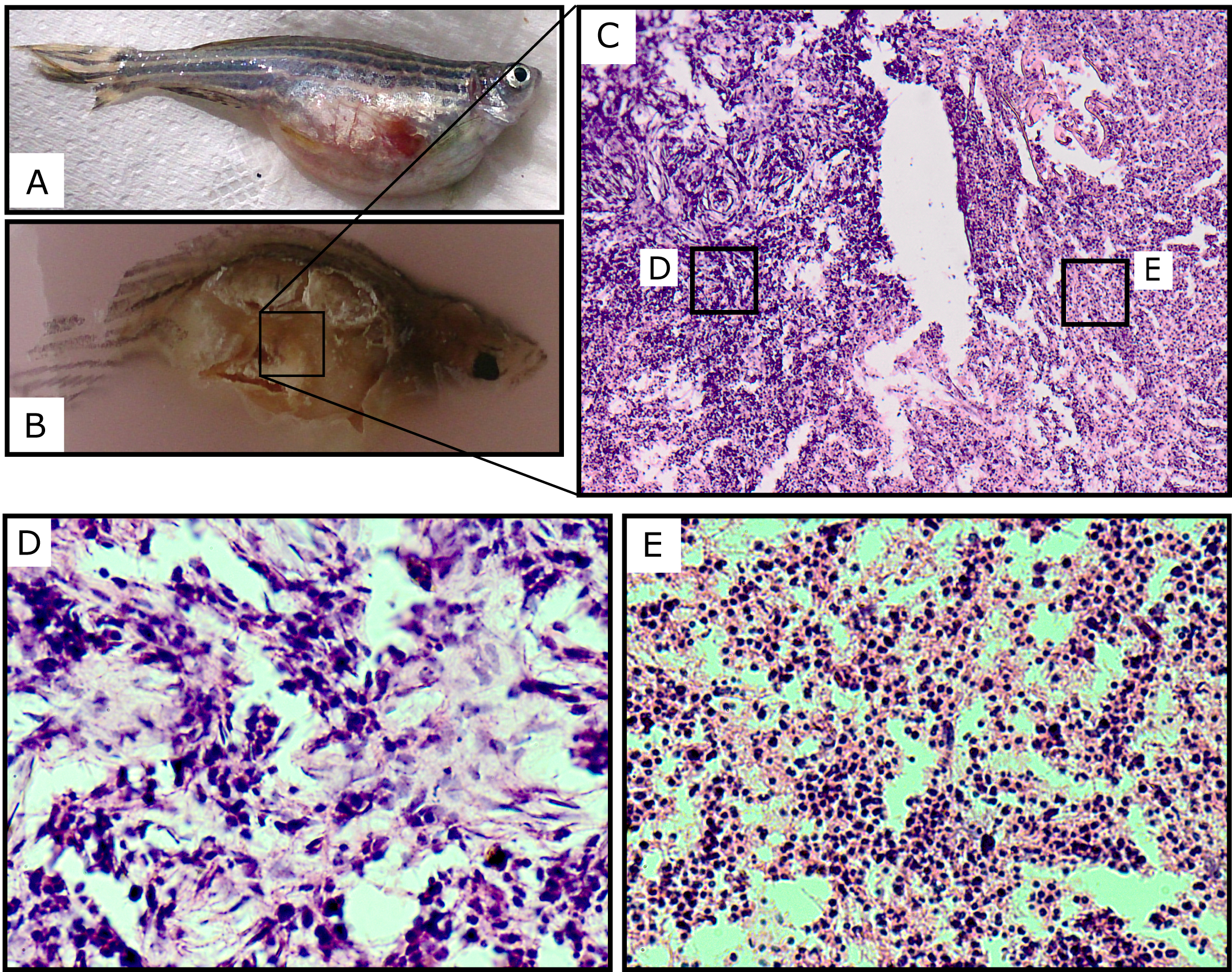

Supplement: S9 Fig — Euthanized 1-year old fish with a distended abdominal cavity (A). Fish were fixed in paraffin blocks (B) and sectioned slides were stained with hematoxylin/eosin for histological examination (C). D and E: enlarged images of sections in C, histological feature of tumors were consistent with the schwannomas as previously demonstrated (36). (TIF) [file pone.0199737.s009.tif]

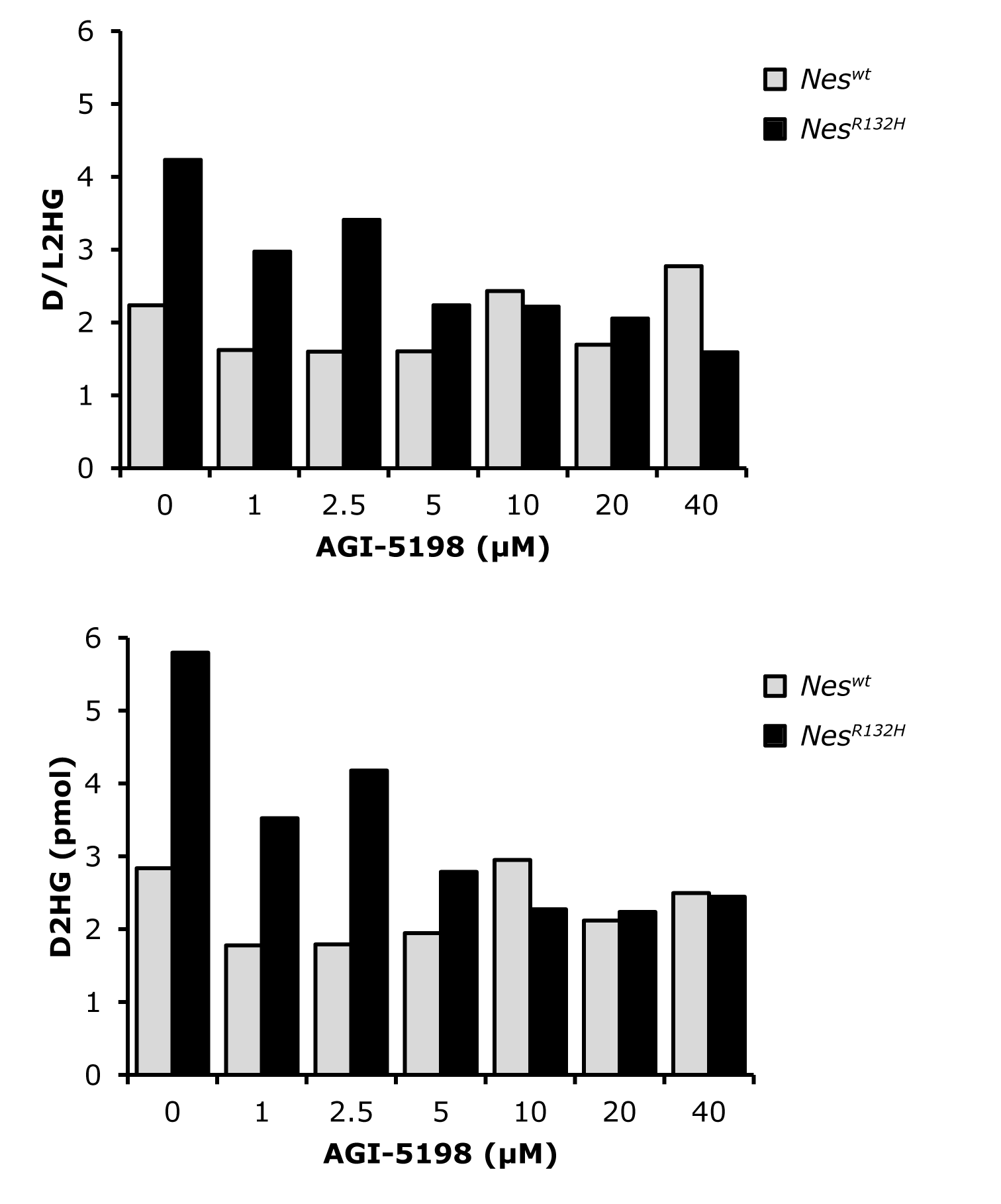

Supplement: S10 Fig — Maximal inhibition is reached at 10μM. (TIF) [file pone.0199737.s010.tif]
