# Supplementary material for: IDH1-mutated transgenic zebrafish lines: An in-vivo model for drug screening and functional analysis
Source: PLoS One. 2018 Jun 28;13(6):e0199737. doi: 10.1371/journal.pone.0199737 (PMC6023169; doi:10.1371/journal.pone.0199737)
Supplement: S1 Table — (PDF) [file pone.0199737.s011.pdf]

Gene	Exon	Primer (5'-3')
IDH1	4	FW- CGACCAAGTCACCAAAGATGC
	4	RV- CCTCAACCCTCTTCTCATCAGG
β-actin	2	FW- CGTGCTGTCTTCCCATCCA
	3	RV-TCACCAACGTAGCTGTCTTTCTG
